# Post acute coronary syndrome Lp(a) testing: To test or not to test?

**DOI:** 10.1016/j.ajpc.2026.101724

**Published:** 2026-08-28

**Authors:** Nathan D. Wong, Jamal S. Rana

**Affiliations:** a Mary and Steve Wen Cardiovascular Division and Center for Global Cardiometabolic Health and Nutrition, University of California, Irvine, California, USA; b Department of Cardiology, The Permanente Medical Group. Kaiser Permanente Oakland Medical Center, California, USA

Lipoprotein(a) [Lp(a)] is a strong independent risk factor for the prediction of future atherosclerotic cardiovascular disease (ASCVD) events in both persons with and without prior ASCVD ([1], [2], [3]) and is among the most powerful genetic predictors of coronary heart disease (CHD) (4). However, recent data on testing rates for Lp(a) remain low, even among those with known ASCVD (5,6), and there remains suboptimal clinician awareness of Lp(a) as a significant risk driver and who should be tested (7). The recent Class 1 recommendation for Lp(a) testing in all adults included in the 2026 ACC/AHA/Multisociety Dyslipidemia Guideline (8), as well as promising new therapies in development (9) have recently motivated increased testing for Lp(a).

What is not clear, however, is the optimal time to test Lp(a), particularly in those with acute coronary syndrome (ACS). Recent data have suggested dynamic changes in Lp(a) following ACS (10,11), which raises questions about the optimal time to test Lp(a) in such patients, and whether a single measurement is reasonable for such patients.

In this issue of the American Journal of Preventive Cardiology, Lattouf and colleagues (12) examine the trajectory in Lp(a) values from serial measurements among 225 patients with ACS over a longer follow-up than previous studies have investigated. The observed increase of 30% after 4–7 days following onset of ACS remained significantly elevated even after 6 months where levels still averaged >20% above baseline, without returning to similar baseline levels until 12 months following ACS. The authors also showed that among those with increases in Lp(a), the baseline Lp(a) was the strongest predictor where higher levels were associated with greater increases. Importantly, they show that those with lower (<50 nmol/L) and higher (≥105 nmol/L) levels at baseline remained relatively stable, but in those at intermediate levels between these cut points, more than a third (37.5%) transitioned to higher risk levels (>105 nmol/L). While the study is of modest size and only certain subsets of patients had follow-up Lp(a) measures available at certain timepoints, the study provides important insight into timing issues for Lp(a) measurement in ACS patients. This study raises some important questions worthy of discussion.

First, when is the optimal time to test Lp(a) among patients with ACS? The case advocating for routine Lp(a) testing at the time of ACS admission finds a parallel in the long-established practice of obtaining a lipid profile on admission. Clinicians have recognized for decades that LDL-cholesterol levels fall measurably within 24–48 h of an acute MI due to the systemic inflammatory response, and that this acute-phase suppression can persist for several weeks, rendering any lipid measurement obtained after that early window an unreliable estimate of the patient’s true baseline. For this reason, guidelines have long recommended that a lipid panel be drawn within the first 24 h of ACS admission—a practice now embedded in standard hospital order sets precisely because it captures the most accurate pre-event lipid status and directly informs the intensity of statin therapy needed for secondary prevention (13). The trajectory in Lp(a) values reported by Lattouf et al. (12) suggest that Lp(a) is subject to an analogous, albeit directionally opposite, acute-phase perturbation: rather than falling, Lp(a) rises substantially in the days following ACS and remains elevated for up to 12 months, meaning that any measurement deferred to a routine outpatient visit within the first year of ACS is likely to overestimate the patient’s true steady-state level, complicating risk stratification and therapeutic decision-making. Just as the lipid profile obtained on admission represents the most actionable snapshot of LDL burden before the acute-phase response distorts the picture downward, an Lp(a) level drawn at the time of ACS admission—before the inflammatory surge drives levels upward—would provide the most reliable baseline for identifying patients who warrant intensified preventive strategies, such as statin treatment and, ultimately, Lp(a)-lowering therapy once such agents demonstrate clinical benefit and become broadly available. Standardizing Lp(a) measurement as part of the ACS admission order set would therefore extend the same clinical logic that has governed lipid testing for a generation, ensuring that this powerful genetic risk factor is captured now when its measurement is both most accurate and most consequential for long-term patient management. Furthermore, utilizing the admission window institutionalizes the test. This ensures that this critical genetic risk metric is captured before the patient navigates the transition to outpatient care, where a reliance on fragmented follow-up and varied primary care clinician awareness frequently leads to missed screening opportunities.

Second, in who among ACS patients should Lp(a) be repeated? The authors suggest those with borderline levels near the threshold for further action who have the greatest likelihood of transitioning to higher risk levels might be considered for re-testing. Others have also shown in a more general patient population that those in the intermediate range are most likely to be reclassified in terms of risk category upon retesting, whereas those who are low or high to begin with are likely to remain in the same risk category (14). The results of Lattouf et al. suggest that Lp(a) levels <50 nmol/L, regardless of when measured following the onset of ACS would likely remain low and below the threshold for further action (12). But for those with higher levels >105 nmol/L, considerations for retesting might depend on how elevated and when measured following the ACS. For instance, someone measured 4–7 days following onset of ACS, if found to have a moderately elevated Lp(a) at 120 nmol/L, could easily transition to level below the threshold for action if retested 6 months later, as the data from Lattouf et al. suggest, whereas someone at much higher levels such as 200 nmol/L would remain elevated to warrant further action regardless of when measured following ACS. Current guidelines call for intensified risk factor reduction for patients with levels ≥50 mg/dL or 125 nmol/, including possible consideration of PCSK9i therapy among ASCVD patients for those needing further LDL-C reduction as has been recently recommended in guidelines (8). Ideally to improve Lp(a) testing rates, such measures could also be considered as future performance measures, comparable to the recent call by both the National Lipid Association and American Society for Preventive Cardiology to bring back lipid profile testing as a performance measure (15).

A third important question this study raises, although much larger studies will be needed to answer this, is whether the substantially elevated risk of recurrent ASCVD events early after ACS is at least in part due to the significant rises in Lp(a) in the first week that the authors observed in their study. While they had done exploratory analyses to examine whether baseline levels or the trajectory in Lp(a) predicted future adverse cardiovascular events, they did not find an association which they explained was likely due to their study being underpowered to do so.

The findings of Lattouf et al. (12) provide important insight into trajectories in Lp(a) that might be expected in the early phase following ACS, supporting a need for standardizing Lp(a) testing at the time of ACS admission and considering retesting those found to be at intermediate levels near the thresholds, where risk reclassification is most likely to occur. Importantly, there is a need for larger studies to investigate whether those with transient increases in Lp(a) shortly after onset of ACS explain part of the early risk of adverse ASCVD outcomes in such patients.

## Author declarations

### Disclosures

Dr. Wong reports research funding through his institution from Amgen, Novartis, and Ionis and is a consultant for Novartis and Lilly. No disclosures reported for Dr. Rana.

## Funding

No funding was received for the preparation of this manuscript.

## CRediT authorship contribution statement

**Nathan D. Wong:** Writing – original draft, Writing – review & editing. **Jamal S. Rana:** Writing – original draft, Writing – review & editing.
